# Tone Mapping of HDR Images via Meta-Guided Bayesian Optimization and Virtual Diffraction Modeling

**DOI:** 10.3390/s25216577

**Published:** 2025-10-25

**Authors:** Deju Huang, Xifeng Zheng, Jingxu Li, Ran Zhan, Jiachang Dong, Yuanyi Wen, Xinyue Mao, Yufeng Chen, Yu Chen

**Affiliations:** 1 Changchun Institute of Optics, Fine Mechanics and Physics, Chinese Academy of Sciences, Changchun 130033, China; huangdeju21@mails.ucas.ac.cn (D.H.); zhengxf@ccxida.com (X.Z.); lijingxu21@mails.ucas.ac.cn (J.L.); zhanran23@mails.ucas.ac.cn (R.Z.); dongjiachang23@mails.ucas.ac.cn (J.D.); wenyuanyi23@mails.ucas.ac.cn (Y.W.); chenyufeng21@mails.ucas.ac.cn (Y.C.); cheny@ccxida.com (Y.C.); 2 Daheng College, University of Chinese Academy of Sciences, Beijing 100049, China; 3 Changchun Cedar Electronics Technology Co., Ltd., Changchun 130103, China

**Keywords:** high dynamic range (HDR), tone mapping, virtual diffraction, meta-learning, Bayesian optimization (BO)

## Abstract

This paper proposes a novel image tone-mapping framework that incorporates meta-learning, a psychophysical model, Bayesian optimization, and light-field virtual diffraction. First, we formalize the virtual diffraction process as a mathematical operator defined in the frequency domain to reconstruct high-dynamic-range (HDR) images through phase modulation, enabling the precise control of image details and contrast. In parallel, we apply the Stevens power law to simulate the nonlinear luminance perception of the human visual system, thereby adjusting the overall brightness distribution of the HDR image and improving the visual experience. Unlike existing methods that primarily emphasize structural fidelity, the proposed method strikes a balance between perceptual fidelity and visual naturalness. Secondly, an adaptive parameter tuning system based on Bayesian optimization is developed to conduct optimization of the Tone Mapping Quality Index (TMQI), quantifying uncertainty using probabilistic models to approximate the global optimum with fewer evaluations. Furthermore, we propose a task-distribution-oriented meta-learning framework: a meta-feature space based on image statistics is constructed, and task clustering is combined with a gated meta-learner to rapidly predict initial parameters. This approach significantly enhances the robustness of the algorithm in generalizing to diverse HDR content and effectively mitigates the cold-start problem in the early stage of Bayesian optimization, thereby accelerating the convergence of the overall optimization process. Experimental results demonstrate that the proposed method substantially outperforms state-of-the-art tone-mapping algorithms across multiple benchmark datasets, with an average improvement of up to 27% in naturalness. Furthermore, the meta-learning-guided Bayesian optimization achieves two- to five-fold faster convergence. In the trade-off between computational time and performance, the proposed method consistently dominates the Pareto frontier, achieving high-quality results and efficient convergence with a low computational cost.

## 1. Introduction

**Figure 1:**
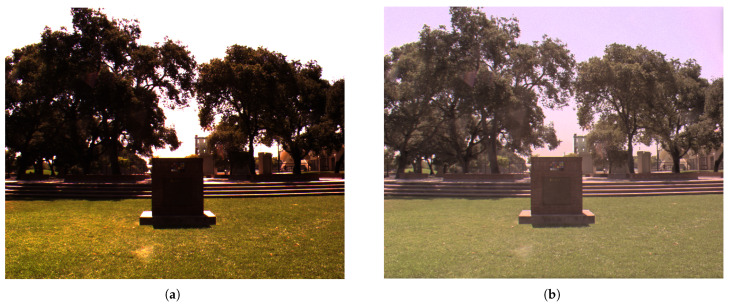
Before-and-after comparison of tone mapping. (**a**) Original image. (**b**) Image after tone mapping.

HDR technology has emerged as a prominent area of research for enhancing visual experiences, as it more accurately replicates the human eye’s perception of natural scene brightness [1,2]. However, mainstream consumer-grade display devices remain constrained due to the inherent limitations of Standard Dynamic Range (SDR) and are thus unable to faithfully preserve the details in extreme shadows or highlights, leading to overexposure or underexposure in certain regions [3,4,5]. For this reason, tone mapping (TM), as a core technology for adapting HDR content to low-dynamic-range displays, has become an essential component of the HDR pipeline [6,7]. Its core task is to transform the luminance values of an HDR image through nonlinear compression into a low-dynamic-range (LDR) representation that can be effectively rendered on standard display devices while preserving structural details and perceptual naturalness as faithfully as possible. In other words, tone mapping seeks to balance dynamic range compression and perceptual fidelity. The before-and-after comparison of the tone mapping is shown in Figure 1.

This technology has significant practical implications across numerous engineering fields. For example, in consumer photography and filmmaking, tone mapping determines the luminance and contrast levels of an image, thereby influencing its perceived visual quality. In autonomous driving and intelligent surveillance, the robustness of the mapping algorithm directly affects object detection and environmental understanding. In medical imaging and remote sensing, mapping quality governs the discernibility of fine details and the accuracy of quantitative measurements. In fields such as advertising displays, virtual reality, and large LED screens, mapping strategies play a crucial role in determining the brightness distribution and visual comfort of display systems.

For this purpose, previous studies can be broadly classified into heuristic-based local and global operators, physically inspired modeling approaches, and data-driven or learning-based methods that have gained increasing prominence in recent years. Each category exhibits distinct advantages and limitations: heuristic-based methods achieve high computational efficiency and intuitive implementation but tend to produce distortions in complex scenes, whereas learning-based approaches often deliver superior perceptual quality on specific datasets yet suffer from limited interpretability and generalization. To balance interpretability and expressive power, an increasing number of studies have sought to incorporate optical principles into tone-mapping frameworks. Such efforts aim to enhance detail preservation and improve the perceptual rationality of luminance distribution through more explicit model constraints.

Additionally, the tone-mapping quality index (TMQI), designed specifically for evaluating tone-mapping performance, comprises two components: structural fidelity and statistical naturalness (NSS). Since the latter is non-differentiable [8], TMQI cannot be directly employed as a loss function for training, and the absence of a well-aligned surrogate loss presents a significant challenge for deep-learning-based tone mapping methods. Because of this, existing methods tend to reward the former and significantly undervalue naturalness, thereby introducing a bias in the TMQI composite score. This bias can result in a visually unnatural appearance due to an insufficient preservation of naturalness, even when the original HDR structural details, such as edges and textures, are retained.

Among the recent advances in the field of image processing, Bahram et al. [9] introduced the concept of “virtual diffraction of light fields,” inspired by the physical characteristics of light propagation. This approach abstracts physical phenomena into mathematical operations, reconceptualizes a digital image as a spatially varying light field, and processes the image by simulating the mathematical behavior of optical methods. By designing a custom-designed virtual phase function, the researchers modulated the phase of the input light field’s spatial spectrum to enhance low-light images. This approach, grounded in physical modeling, represents a novel research paradigm for image processing.

Based on these observations, we propose a new method for image tone mapping that integrates meta-learning, a psychophysical model, Bayesian optimization, and virtual diffraction into a unified framework. The main contributions of this work are summarized as follows.

(1) Our method integrates virtual diffraction—a mathematical construct abstracted from physical principles—into the tone mapping pipeline. From the frequency domain perspective, the HDR image undergoes phase reconstruction using a phase modulation function. By controlling the parameters of virtual diffraction, precise adjustments of image details and contrast can be achieved. Simultaneously, the Stevens power law [10] is incorporated to model the human visual system’s nonlinear perception of luminance, thereby establishing a tone mapping model that effectively enhances structural fidelity and the visual quality of the resulting LDR image.

(2) To determine the model parameters, we develop an efficient and adaptive optimization framework. Based on the active learning principle inherent to Bayesian optimization, a fully automated parameter tuning mechanism is employed for key variables in the tone-mapping model. Since TMQI is non-differentiable and cannot be optimized using gradient-based methods, Bayesian optimization enables direct optimization by modeling uncertainty through probabilistic inference without relying on surrogate losses. This facilitates the approximation of the global optimum with fewer evaluations, significantly improving optimization efficiency. Furthermore, since Bayesian optimization does not rely on gradients, the optimization process can simultaneously account for both fidelity and NSS, thereby addressing the distortions arising from the disproportionate emphasis on fidelity over naturalness in existing methods.

(3) To enhance the model’s generalization and task adaptability, we propose a task distribution-oriented meta-learning framework. A meta-feature space is constructed by extracting statistical features from input images and is integrated with a clustering mechanism to enable task-level grouping and episodic training. On this basis, a meta-learner equipped with a gating mechanism is employed for rapid parameter initialization and fast adaptation through a limited number of updates on new images, thereby improving the robustness and overall performance of the proposed method across HDR images exhibiting diverse content and characteristics, under the same computational budget. Furthermore, the transfer of prior knowledge in meta-learning accelerates convergence and alleviates the cold-start issue encountered in conventional Bayesian optimization.

The remainder of this paper is organized as follows: Section 2 reviews existing research related to tone mapping. Section 3 presents the physical foundations of virtual light field diffraction. Section 4 provides a detailed description of the proposed methodology, including the construction of the phase modulation function, the formulation of the tone-mapping model, and the parameter optimization strategy. Section 5 presents the experimental results in detail, covering ablation studies, performance evaluation, and a comparison with state-of-the-art algorithms. Finally, Section 6 concludes the paper with a comprehensive summary.

## 2. Related Work

The development of tone-mapping operators (TMOs) demonstrates the convergence of perceptual science, image processing, and machine learning. Traditional TMOs are primarily based on the human visual system (HVS) and physiological or psychophysical models [11,12]. Some approaches [13,14,15,16,17,18] employ uniform mapping functions to adjust pixel luminance. For example, Drago et al. [16] proposed an adaptive logarithmic mapping strategy that compresses the dynamic range based on differences in scene luminance. Kim et al. [17] adjusted the luminance distribution of an image by modeling the nonlinear transformation of the HVS in terms of luminance sensitivity. Although these methods are theoretically aligned with human perception, physically interpretable, computationally efficient, and easy to implement, they exhibit significant limitations in preserving local details and aligning with subjective perception. In addition, histogram-based redistribution strategies are widely used for contrast enhancement and detail preservation. Examples include hybrid histogram models incorporating linear and balanced mappings [19], constraint-based methods in the perceptual domain, and optimized mappings that utilize non-uniform binning tailored to bright and dark regions [20]. While these histogram-based methods are simple and efficient, they are prone to excessive contrast stretching in regions of similar luminance. To address these limitations, some approaches employ local operators [21,22,23,24] that leverage contextual cues from neighboring pixels to perform tone mapping and compress the dynamic range. Clustering-based strategies have also been proposed to group luminance values and represent localized luminance distributions using techniques such as K-means or Gaussian mixture models [25,26]. Although local operators, including clustering strategies, enable targeted processing of localized regions and mitigate the limitations of global methods by segmenting image content, they often suffer from high computational complexity and are prone to halo artifacts, posing challenges in balancing contrast preservation and artifact suppression. Qiu et al. [27] recently introduced a multi-peak S-shaped tone-mapping curve that integrates the human visual system’s luminance adaptation mechanism with histogram analysis. The method partitions grayscale intervals based on the peak–valley structure of the histogram, assigning distinct tone curves to each segment. This reduces compression in dense grayscale regions, enhances it in sparse regions, and ultimately improves visual quality. While the method shows notable improvement, its effectiveness remains limited, indicating potential for further enhancement.

In recent years, deep learning has offered novel research paradigms in the field of tone mapping, enabling data-driven frameworks to model the intricate transformation between HDR and low-dynamic-range (LDR) images. For example, DeepTMO [28] employs a conditional generative adversarial network (CGAN) to directly generate high-resolution LDR images that retain HDR information. Cao et al. [29] propose an adversarial tone-mapping operator aimed at enhancing the visual fidelity of LDR images through adversarial training. TMO-Net [30] formulates the HDR mapping task as an image enhancement problem and leverages large-scale low-light image datasets to enhance network generalization. Hu et al. [31] developed a multi-scale convolutional network capable of simultaneously handling tone mapping and image enhancement tasks. These methods have the advantage of automatically capturing complex nonlinear mappings while minimizing manual parameter tuning and heuristic design. However, their performance is heavily reliant on large-scale, high-quality HDR-LDR paired datasets. Such datasets are typically scarce and challenging to acquire due to costly annotations and substantial subjective variability. To address this limitation, current research primarily follows two directions. The first involves generating candidate LDR images via dynamic range compression of HDR images using existing tone-mapping algorithms, followed by utilizing the TMQI [32] to quantitatively select optimal outputs. However, this method is inherently constrained due to the performance bottlenecks of existing algorithms, making it challenging to overcome the limitations of established frameworks. The second approach draws inspiration from low-light image enhancement and involves training on pairwise datasets comprising bright and dark scenes. Nevertheless, the significant differences in light distribution and physical characteristics between HDR images and low-light scenes may limit the model’s cross-domain generalization capabilities.

To address the challenge of insufficient training data, several studies have explored semi-supervised and unsupervised learning strategies. For instance, Zhang et al. [33] proposed a semi-supervised deep tone-mapping network trained using limited paired data and abundant unlabeled LDR images, thereby improving the model’s robustness. Guo et al. [8] leveraged image quality metrics as supervisory signals within a semi-supervised framework, incorporating segmentation priors to improve image content perception. Vinker et al. [34] employed an unsupervised strategy to train a mapping model on unpaired HDR images via adversarial learning, enabling the network to autonomously learn luminance mapping rules, thereby eliminating the reliance on labeled data, reducing paired sample requirements, and expanding the applicability of tone mapping algorithms. More recently, Cao et al. [35] proposed an unsupervised method based on contrastive learning, which extracts luminance and contrast features through a novel latent representation, measures inter-image similarity, and utilizes a spatial feature enhancement module to facilitate non-local information exchange for HDR tone mapping. Although these approaches partially alleviate the reliance of supervised learning on paired datasets, they still encounter challenges such as unstable training, slow convergence, and significant output variability. In particular, in the absence of real HDR semantic supervision, the model often struggles to capture patterns of local contrast and texture variation and still suffers from issues such as edge blurring and local structural degradation.

In summary, although classical TMOs, histogram-based approaches, local operators, and HVS-inspired deep learning methods have each made notable contributions to dynamic range compression, they still exhibit evident limitations in detail preservation, consistency between subjective perception and objective metrics, the scarcity of training data, and adaptive parameter optimization.

## 3. Physical Foundations

The propagation of light in free space follows the scalar diffraction theory. Let the input light field be $E_{i}\left(x,y,0\right)$ and its spatial spectrum be the following:

(1) $${\tilde{E}}_{i}\left(f_{x},\;f_{y},0\right)=\int_{-\infty }^{\infty }\int_{-\infty }^{\infty }E_{i}\left(x,y,0\right)e^{-i2\pi \left(f_{x}x+f_{y}y\right)}dxdy$$

where $f_{x}$ and $f_{y}$ denote the spatial frequencies in the *x* and *y* directions, respectively. After propagating a distance, *z*, the spatial spectrum undergoes a phase shift described as follows:

(2) $${\tilde{E}}_{0}\left(f_{x},\;f_{y},z\right)={\tilde{E}}_{i}\left(f_{x},\;f_{y},0\right)e^{-i\phi (f_{x},\;f_{y})}$$

The spectral phase accumulation satisfies the following: $\phi \left(f_{x},\;f_{y}\right)=\frac{2\pi^{2}z}{k_{0}}(f_{x}^{2}\;+\;f_{y}^{2})$ (Fresnel approximation), where $k_{0}$ is the wave number, denoted as $k_{0}=\frac{2\pi }{\lambda }$, which reflects the quadratic phase growth property of physical diffraction, i.e., the diffraction phase increases monotonically with spatial frequency. The propagating signal after phase accumulation can be expressed as follows:

(3) $$E_{o}\left(x,y,z\right)=\int_{-\infty }^{\infty }\int_{-\infty }^{\infty }{\tilde{E}}_{i}\left(f_{x},\;f_{y},0\right)e^{-i\phi \left(f_{x},\;f_{y}\right)}\cdot e^{i2\pi \left(f_{x}x\;+\;f_{y}y\right)}df_{x}df_{y}$$

**Figure 2:**
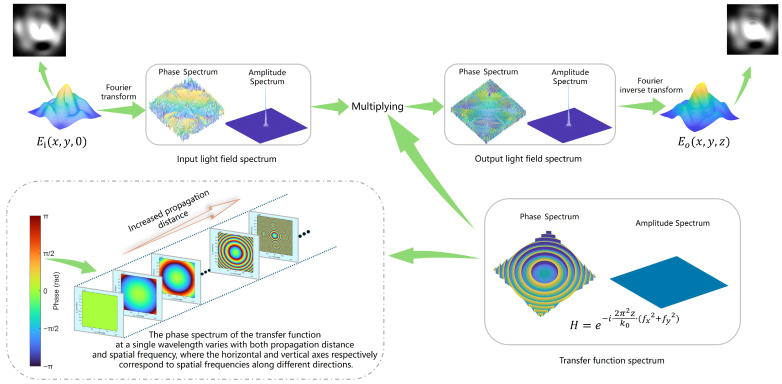
Schematic diagram of light-field diffraction in free space (Fresnel approximation).

That is, $E_{o}\left(x,y,z\right)=\mathrm{IFT}\left\{{\tilde{E}}_{i}\left(f_{x},\;f_{y},0\right)e^{-i\phi \left(f_{x},\;f_{y}\right)}\right\}$, reflecting the fact that the output light field after propagation over a distance of *z* is a Fourier inverse transformation of the spatial spectrum of the input light field after a phase shift. The overall physical process is illustrated in Figure 2.

Since the processing object is a digital image and the light field is pixelized, the processing target transitions from the continuous domain to the discrete domain. When the spatial sampling interval Δx, Δy satisfies the Nyquist condition, the continuous field E(x, y) is discretized as E(m, n), where $m=\frac{x}{\Delta x}$, $n=\frac{y}{\Delta y}$. The spatial frequencies $\left(f_{x},\;f_{y}\right)$ are discretized as $\left(\frac{k_{m}}{M\cdot \Delta x},\frac{k_{n}}{N\cdot \Delta y}\right)$, where $k_{m}=0,1,\ldots ,M-1$; $k_{n}=0,1,\ldots ,N-1$. At this point, the discretized output light field is expressed as follows:

(4) $$\begin{array}{cc} E_{o}\left(m,n\right) & =\frac{1}{MN}\left\{\sum_{k_{m}=0}^{M-1}\sum_{k_{n}=0}^{N-1}\left\{\left[\sum_{m=0}^{M-1}\sum_{n=0}^{N-1}E_{i}\left(m,n\right)e^{-j2\pi (\frac{k_{m}m}{M}+\frac{k_{n}n}{N})}\right]\;e^{-i\phi (k_{m},k_{n})}\right\}\;e^{j2\pi (\frac{k_{m}m}{M}+\frac{k_{n}n}{N})}\right\}\\ \; & =\mathrm{IDFT}\{\mathrm{DFT}\left[E_{i}\left(m,n\right)\right]\cdot e^{-i\phi (k_{m},k_{n})}\} \end{array}$$

In the physical process of diffraction, the phase change obeys a well-defined physical law. However, with the introduction of the concept of virtual diffraction, the light field becomes discretized, allowing the output to be modulated by an arbitrary phase function. Therefore, the design of the phase modulation function is of critical importance in image processing.

## 4. Method

**Figure 3:**
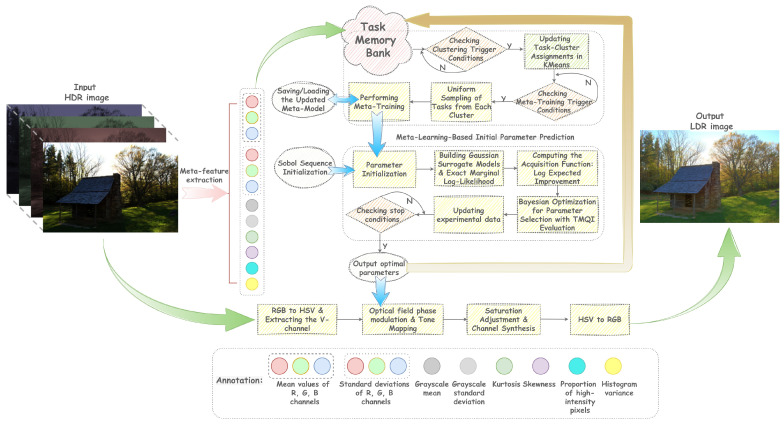
Overall framework diagram of tone mapping.

This paper employs frequency-domain phase modulation of light fields and the Stevens power law, derived from psychophysics, as its core computational operators. The overall workflow is as follows: first, feature vectors capturing key attributes such as luminance distribution and local contrast are extracted from the input HDR image to guide the meta-learner in producing initial parameter estimates. Subsequently, taking these initial estimates as the starting point, the luminance channel undergoes a Fourier transform, phase modulation, and inverse transformation under a parameterized frequency-domain operator to generate a preliminary LDR luminance map. The resulting luminance map is then converted into final pixel intensities through coherent detection and a nonlinear transformation governed by the Stevens power law. This luminance map is subsequently fused with the chrominance channels to produce a candidate LDR image. Finally, an iterative parameter optimization is performed using a Bayesian optimization strategy that employs a Gaussian process as the surrogate model and integrates an information-driven acquisition function. At each iteration, a candidate image is generated through the aforementioned forward pipeline and evaluated using the TMQI metric. This process is repeated until convergence or until a predefined stopping criterion is satisfied, yielding the optimal parameters and corresponding results. The complete workflow of the proposed framework is illustrated in Figure 3.

### 4.1. Phase Modulation Function Modeling

In tone mapping, the primary objective is to address the dynamic range discrepancy between HDR content and LDR display systems. Specifically, tone mapping adjusts the luminance distribution of an image by compressing excessive luminance variations while preserving image detail. Low-frequency information, which constitutes the dominant component of an image, encapsulates the approximate content distribution. Therefore, the design of the phase modulation function should primarily target the low-frequency components of the image. Moreover, the phase shift of the modulated optical field should be constrained within a small range, analogous to the phenomenon of near-field diffraction.

In this paper, we use a Gaussian function with mean-0 variance *T* as $\phi \left(k_{m},k_{n}\right)$:

(5) $$\phi \left(k_{m},k_{n}\right)=S\cdot e^{-\frac{k_{m}^{2}+k_{n}^{2}}{T}}$$

The selection of the Gaussian function is grounded in fundamental theoretical principles in the field of signal processing and its practical advantages in subsequent optimization processes. Theoretically, the Gaussian function exhibits infinite differentiability, thereby ensuring the highest possible smoothness of the transfer function in the frequency domain. This smoothness is essential for preventing ringing artifacts and other undesirable discontinuities in the spatial domain—issues that commonly arise from the inverse Fourier transforms of functions exhibiting abrupt transitions, such as linear or truncated polynomial functions. Furthermore, its characteristic bell-shaped curve provides an optimal structure for selective frequency attenuation, imposing a substantial and nearly uniform phase shift on the desired low-frequency components while asymptotically approaching the original phase at higher frequencies. This property effectively preserves the fine details and textures essential for tone mapping applications. From a practical standpoint, the functional form is notably compact, requiring only two physically interpretable parameters—the modulation gain (*S*) and the variance (*T*)—for complete characterization. This parametric simplicity significantly enhances the efficiency of the Bayesian optimization framework by reducing the dimensionality of the search space and accelerating convergence toward a stable global optimum.

Then, the phase modulation function is as follows:

(6) $$H=e^{-i\cdot S\cdot e^{-\frac{k_{m}^{2}+k_{n}^{2}}{T}}}=\cos\left(S\cdot e^{-\frac{k_{m}^{2}+k_{n}^{2}}{T}}\right)-isin\left(S\cdot e^{-\frac{k_{m}^{2}+k_{n}^{2}}{T}}\right)$$

where *S* is the modulation gain, similar to the propagation distance in physical diffraction, which controls the overall phase offset. *T* determines the cutoff frequency of the modulation. When $k_{m}^{2}+k_{n}^{2}\gg T$, corresponding to the high-frequency component, the phase offset is attenuated exponentially, and *H* approaches 1 without any modulation; when $k_{m}^{2}+k_{n}^{2}\ll T$, corresponding to the low-frequency component, the phase offset approaches *S*.

The original image contains only a real component, with an implicitly zero imaginary part, and frequency-domain phase modulation is mathematically equivalent to introducing asymmetric phase shifts across spatial frequencies in the wavefront. This operation alters the coherence characteristics of the light field, resulting in a reconstructed spatial-domain distribution comprising both real and imaginary components. The output thus becomes a complex amplitude distribution that describes the complete wavefront, directly capturing the changes in spatial interference patterns introduced via phase modulation.

**Figure 4:**
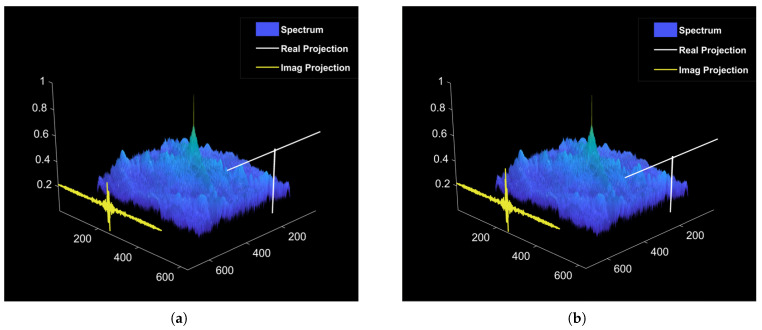
Energy conversion diagram for phase modulation. (**a**) Before phase modulation. (**b**) After phase modulation.

According to Parseval’s theorem, the total energy of the optical field is conserved in both the time and frequency domains, and phase modulation does not alter the spectral amplitude $\left(|{\tilde{E}}_{o}|=|{\tilde{E}}_{i}|\right)$. Therefore, phase modulation does not change the total energy of the light field, but redistributes the projection of the energy on the real part and the imaginary part. Under a small phase offset, the extreme cases can be considered: near-zero frequency, $Re\left({\tilde{E}}_{o}\right)\approx {\tilde{E}}_{i}cosS$, indicating slight attenuation in the real component, and $Im\left({\tilde{E}}_{o}\right)\approx -{\tilde{E}}_{i}sinS$, indicating a substantial increase in the imaginary component. In the ultra-high-frequency (UHF) region, $Re\left({\tilde{E}}_{o}\right)\approx {\tilde{E}}_{i}$, implying that the real component remains nearly unchanged, while $Im({\tilde{E}}_{o})\approx 0$, meaning the imaginary component is negligible. Consequently, the energy attenuation observed in the low-frequency region of the real component corresponds to a transfer toward the low-frequency region of the imaginary component, whereas the energy in the high-frequency domain remains nearly unchanged. The energy conversion diagram is illustrated in Figure 4.

### 4.2. Constructing the Tone Mapping Operator

To ensure hue consistency, the luminance and chrominance components of the input image must be separated. Due to the independence of hue in HSV space and the stability of its luminance information, which aligns more closely with human visual perception than the HSL space, the HSV color space is adopted in this study. This color space is particularly suitable for applications requiring minimized luminance influence while preserving color fidelity. This study primarily processes the S and V channels. First, the luminance channel of the input image is extracted, and a bias term b is introduced. This bias term introduces a direct current (DC) component representing background light into the light field, thereby enhancing the zero-frequency component of the spectrum, which improves numerical stability and reduces computational noise. The image is transformed from the spatial domain to the frequency domain using the fast Fourier transform (FFT) and then multiplied by the phase modulation function H to obtain the modulated spatial spectrum, as given via the following expression:

(7) $${\tilde{E}}_{o}\left(k_{m},k_{n}\right)=\mathrm{FFT}\left(E_{i}^{V-Channel}\left(m,n\right)+b\right)\cdot e^{-i\cdot S\cdot e^{-\frac{k_{m}^{2}+k_{n}^{2}}{T}}}$$

Subsequently, the modulated spectrum is transformed back into the spatial domain via the inverse fast Fourier transform (IFFT), followed by coherent detection of the resulting complex-valued signal, thereby replacing the original image with the extracted phase information. The expression is shown in Equations (8) and (9).

(8) $$E_{o}\left(m,n\right)=\mathrm{IFFT}\left({\tilde{E}}_{o}\left(k_{m},k_{n}\right)\right)$$

(9) $$V_{f}\left(m,n\right)=\tan^{-1}\left(G\cdot \frac{\mathrm{imag}\left(E_{o}\left(m,n\right)\right)}{\mathrm{real}\left(E_{o}\left(m,n\right)\right)}\right)$$

In the small-phase case, $V_{f}\left(m,n\right)$ can be approximated as follows:

(10) $$V_{f}\left(m,n\right)\approx G\cdot \frac{\mathrm{imag}(E_{o}\left(m,n\right))}{\mathrm{real}(E_{o}\left(m,n\right))}$$

**Figure 5:**
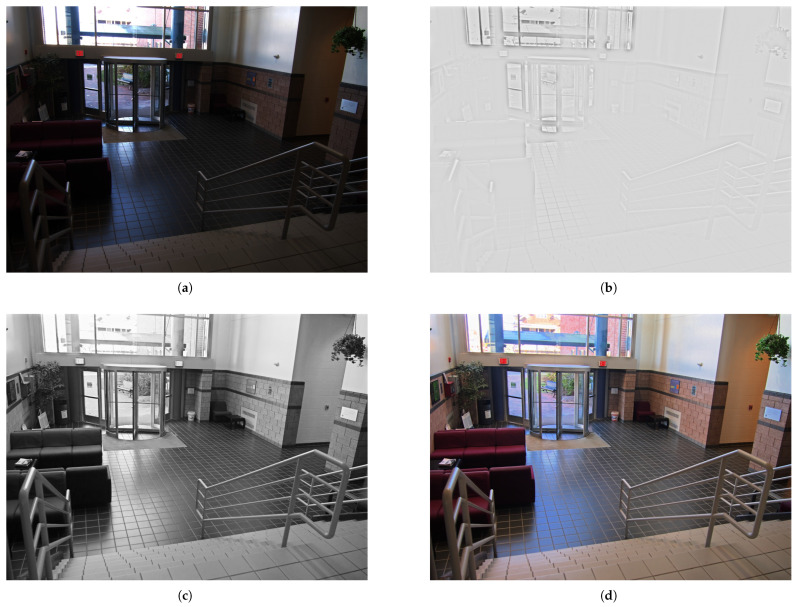
Results of phase modulation and coherent detection. (**a**) Original image. (**b**) V channel after the first coherent detection. (**c**) V channel after the second coherent detection. (**d**) RGB image after channel synthesis.

Specifically, *G* represents the phase activation gain, serving to scale the phase change and enhance phase sensitivity by amplifying the ratio between the imaginary and real components of the complex signal. Subsequently, the phase information is recovered from the real and imaginary components using the arctangent function. The real part of the complex signal approximates the original optical field, while the imaginary part encodes the phase modulation component introduced via virtual diffraction. The value of $V_{f}\left(m,n\right)$ is directly proportional to the imaginary part of $E_{o}\left(m,n\right)$ and inversely proportional to the spatial frequency of the original image, $E_{i}^{V-Channel}$. This relationship enables contrast reduction in the low-frequency range and enhancement in the high-frequency range, thereby achieving dynamic range compression while preserving fine details and enhancing subtle structural features, as illustrated in Figure 5b.

Then, the original image is used as a reference light field and combined with $V_{f}\left(m,n\right)$ to construct a new complex amplitude. The phase of the new light field is then extracted:

(11) $$E_{\mathrm{tot}}\left(m,n\right)=E_{i}^{V-\mathrm{Channel}}\left(m,n\right)+i\cdot V_{f}\left(m,n\right)$$

(12) $$V_{s}\left(m,n\right)=\mathrm{Norm}\left(\tan^{-1}\left(\frac{V_{f}\left(m,n\right)}{E_{i}^{V-\mathrm{Channel}}\left(m,n\right)}\right)\right)$$

Among these, Norm refers to “Min-Max” normalization. During the second coherent detection process, the original light field is regarded as the “strong reference field.” $V_{f}\left(m,n\right)$ is considered weakly phase-modulated light, which contains phase perturbations introduced via the “diffraction component.” Through the second coherent detection and normalization, the weak signal is further amplified, whereas the strong signal is correspondingly compressed. Additionally, by adjusting parameters such as *S*, *G*, *b*, and *T*, the overall characteristics of the light field can be modulated. From a mathematical perspective, the inverse tangent function maps linear phase differences to nonlinear responses, leading to saturation in extreme scenes and linear enhancement in moderate ones, thereby effectively compressing the image’s dynamic range. Figure 5c presents the V channel after the second coherent detection, and Figure 5d illustrates the RGB image following channel synthesis.

However, since HDR images typically exhibit a significantly high dynamic range, the aforementioned operation alone is insufficient to achieve the desired tone mapping effect. Therefore, this paper proposes an additional dynamic range adjustment. Leveraging the relationship between stimulus intensity and subjective perception described by Stevens’s power law in psychophysics, the nonlinear luminance response of the human visual system is simulated to re-adjust the luminance distribution of HDR images. The final output of the luminance channel is given as follows:

(13) $$V_{o}\left(m,n\right)={\left(A\cdot V_{s}\left(m,n\right)\right)}^{\gamma }$$

Here, *A* represents the scale factor, and γ denotes the power index of luminance perception. Additionally, due to the computational characteristics of the HSV color space, luminance adjustments may indirectly influence saturation. Therefore, the saturation must also be adjusted. The final output of the saturation channel is given as follows:

(14) $$S_{o}\left(m,n\right)=B\cdot S_{i}\left(m,n\right)$$

where $S_{i}\left(m,n\right)$ is the input image saturation, and *B* is the saturation shrinkage factor.

### 4.3. Parameter Optimization and Adaptive Output

According to the method described in the previous subsection, different HDR images can be tone-mapped by adjusting seven parameters: *S*, *G*, *b*, *T*, *A*, *B*, and γ. However, distinct image features often correspond to substantially different optimal parameter values. Manual tuning is not only inefficient but also highly subjective, lacking a unified optimization criterion. Therefore, it is crucial to adopt an automated optimization approach to identify the optimal parameters. This process can be formalized as an optimization problem, as expressed mathematically below:

(15) $$\begin{array}{c} \theta^{*}=\underset{\theta \in D}{\arg\max}L\left(\theta \right):=\mathrm{TMQI}\left(I_{HDR};T\left(I_{HDR};\theta \right)\right)\\ \;s.t.\;\theta ={\left[\theta_{1},\theta_{2},\ldots ,\theta_{7}\right]}^{T}={\left[S,G,b,T,A,\;B,\gamma \right]}^{T}\\ D=\prod_{i=1}^{7}\left[\theta_{i}^{\min},\theta_{i}^{\max}\right]\subset R^{7} \end{array}$$

where θ is the hyperparameter vector of tone mapping, and $\theta^{*}$ is the optimal hyperparameter vector, D is the search space, $I_{HDR}$ denotes the input HDR image, T is the proposed tone mapping operator, L(θ) is the overall target generalization, and $\left[\theta_{i}^{\min},\theta_{i}^{\max}\right]$ is the range of intervals for the ith parameter.

However, for this optimization problem, various types of methods exhibit various limitations. Grid search becomes infeasible in a seven-dimensional parameter space due to the combinatorial explosion. Although stochastic search avoids this issue, its uninformed sampling strategy may result in slow convergence, poor robustness, and difficulty in reproducing results. Heuristic algorithms, which rely on random search or group collaboration mechanisms, typically require a large number of objective function evaluations to explore the solution space. This leads to inefficient sample utilization, limited applicability in scenarios with high evaluation costs, and an absence of rigorous convergence guarantees. In contrast, Bayesian optimization, grounded in a probabilistic agent model with an active learning mechanism, significantly enhances sample utilization efficiency by balancing exploration and exploitation to approximate a globally optimal solution with a limited number of evaluations. Moreover, while the performance of heuristic methods is highly sensitive to parameter settings and lacks formal convergence guarantees, Bayesian optimization—leveraging a probabilistic modeling framework—can quantify uncertainty in the objective function and guide the search direction using an acquisition function, thereby offering stronger theoretical guidance. However, traditional Bayesian optimization still relies on random or uniform sampling during initialization, treats each new task independently, fails to leverage shared knowledge from historical tasks, and lacks prior knowledge of potentially optimal regions. As a result, multiple iterations are needed to correct the agent model, thereby significantly prolonging the convergence time.

Therefore, this article proposes a Bayesian optimization method guided by a meta-learning mechanism for parameter optimization, with TMQI as the objective function, and outputs the corresponding optimal parameters and tone-mapped output image when the objective function reaches its maximum value. Through meta-learning, the unbiased exploration process is transformed into an experience-driven, directed search process, enabling the transfer of optimal strategies across similar tasks and reducing the overhead of repeated search.

The proposed methodology was carried out in three stages.

#### 4.3.1. Meta-Feature Extraction

Through meta-feature extraction, the key statistical information of an image is encoded into low-dimensional vectors that describe image attributes such as brightness, contrast, and color distribution. These features are used as inputs to the meta-learning model, enabling the model to rapidly capture the visual characteristics of a new image and thereby predict the tone-mapping parameters most suitable for the given image. The primary functions of meta-features within the meta-learning model are as follows: providing global statistical information about the image to narrow the parameter search space, and avoiding the direct use of raw pixel data, thereby reducing the model’s sensitivity to local noise.

In this paper, a total of 12 meta-features are selected, including the mean and standard deviation of the RGB channels, luminance mean, luminance standard deviation, luminance kurtosis, luminance skewness, the proportion of highlight pixels, and histogram variance. The mean and standard deviation of the RGB channels represent the average luminance and luminance contrast for each channel, respectively, while the luminance mean and standard deviation indicate the overall brightness level and contrast of the image. Luminance kurtosis is used to quantify the peakedness or flatness of the luminance distribution, and luminance skewness is used to assess its symmetry—left skewness suggests a predominance of darker regions, whereas right skewness implies that brighter regions predominate. The proportion of highlight pixels indicates the extent of bright areas in the image, and histogram variance measures the distributional uniformity of luminance values.

Meta-features capture three main types of statistical characteristics of an image across multiple dimensions. Among them, color distribution is primarily used to represent the color balance and contrast of the image, while luminance statistics represent its overall luminance distribution. The proportion of highlight pixels and the variance of the luminance histogram offer additional complementary information regarding extreme luminance regions and the uniformity of their distribution. This enables the meta-learning model to more effectively leverage historical optimization knowledge and improve the efficiency of parameter search in new tasks.

#### 4.3.2. Meta-Learning Training

In the meta-learning phase, this paper draws on the basic idea of Model-Agnostic Meta-Learning (MAML) [36] and incorporates a dynamic gating mechanism with a multi-step parameter adaptation strategy. The overall framework consists of two components: a meta-learner and a meta-trainer. The meta-learner adjusts feature weights through a dynamic gating mechanism and leverages gradient descent to achieve rapid task adaptation, while the meta-trainer utilizes an optimization strategy that combines inner and outer loops to update meta-parameters across tasks. Given that meta-learning aims to accelerate the convergence process of Bayesian optimization and reduce computational resources and time costs, the meta-learning model used here should maintain a simple structure.

The specific implementation is as follows: the extracted meta-features are normalized and then fed into a task memory module to check whether clustering conditions are met, after which historical task features are grouped through clustering. At the task management level, this article introduces a K-Means clustering mechanism to enable a hierarchical sampling of historical task features, thereby ensuring the coverage of task distribution diversity and reducing overfitting risks due to distributional shifts.

**Figure 6:**
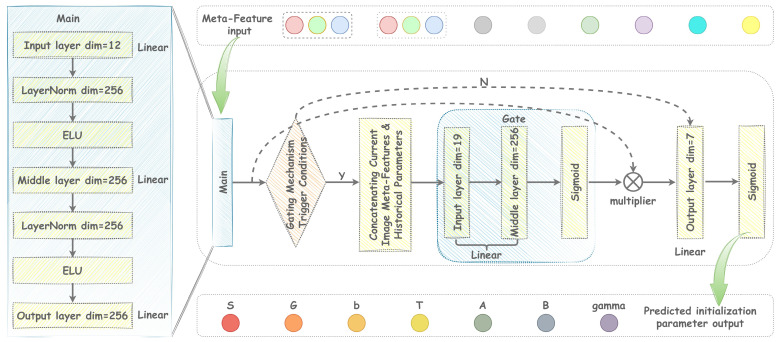
Block diagram of the meta-learner structure.

The network structure of the meta-learner is illustrated in Figure 6 and primarily consists of a gating network and a backbone network. The gating network concatenates the current input features with the parameters predicted in the previous inner-loop iteration and generates gating coefficients within the range of [0, 1] via a Sigmoid activation function, serving as a soft gating mechanism for feature selection. The backbone network enhances parameter space modeling via multilayer linear transformations, ELU-based nonlinear activations, and layer normalization, ultimately extracting high-order features and outputting a normalized seven-dimensional parameter vector.

During prediction, the gating mechanism dynamically modulates the feature weights output via the backbone network. When historical results are available, the gating network concatenates the current input with past results to generate a gating mask, thereby endowing the prediction process with temporal memory and improving the model’s representation of the task’s dynamic features. The forward propagation process of the model can be formalized as follows:

(16) $$\begin{array}{c} h_{\mathrm{main}}=f_{\mathrm{main}}\left(x\right)=ELU\left(\;\mathrm{LayerNorm}\;\left(W_{2}\cdot ELU\left(\;\mathrm{LayerNorm}\;\left(W_{1}x+b_{1}\right)\right)\right)\right)\\ g_{t}=\sigma \left(W_{g}\left[x;p_{t-1}\right]+b_{g}\right)\\ h_{\mathrm{gate}}=h_{\mathrm{main}}⊙g_{t}\\ p_{t}=\sigma \left(W_{o}h_{\mathrm{gate}}+b_{o}\right) \end{array}$$

where $W_{1}\in R^{\left(256\;\times \;12\right)}$, $W_{2}\in R^{\left(256\;\times \;256\right)}$ is the main network weight matrix, $W_{g}\in R^{\left(256\;\times \;19\right)}$ is the gated network weights, $W_{o}\in R^{\left(7\;\times \;256\right)}$ is the output network weights, σ is the Sigmoid activation function, and ⊙ denotes the Hadamard product. $p_{t-1}$ denotes the parameters of the previous step, $⊙\left[x;p_{t-1}\right]\in R^{\left(19\;\times \;1\right)}$ denotes the splicing vector of the current input with the previous step parameters, and $p_{t}$ is the normalized prediction parameter of the output.

During training, the inner loop generates task-specific parameters through multi-step gradient descent, while the outer loop evaluates the meta-losses based on the adapted parameters, thereby updating the initial parameters of the meta-model. The gradient computation graph is preserved in the inner loop, thereby supporting second-order derivative computation, as well as backward gradient propagation, which accurately captures the sensitivity of the meta-parameters to the task adaptation process. Furthermore, the incorporation of a task memory buffer and a K-means clustering mechanism optimizes the task-sampling strategy, thereby enhancing the model’s ability to generalize over complex task distributions through clustering analysis of historical task features. After obtaining a valid output, the output is inverse-normalized to a fixed range within the search space and subsequently passed to the Bayesian optimization module for initialization.

#### 4.3.3. Bayesian Optimization

The initialization phase of Bayesian optimization is a critical determinant of overall optimization performance, and the quality of its design directly influences the initial accuracy, optimization efficiency, global convergence, and resource utilization of the surrogate model. As a sequential optimization method based on a probabilistic surrogate model, its initialization stage provides an initial approximation of the objective function’s topology by appropriately selecting an initial set of sample points, thereby constructing a reliable prior distribution to guide the subsequent acquisition function. Therefore, in this work, the initialization process is guided by the predicted outputs of the meta-learning model. Upon the input of the first image, the meta-learning model lacks predictive capability and is, therefore, initialized with a random sequence. After 50 tasks, the meta-learning model generates a usable output, and this output is incorporated into the method described in Section 4.2. The TMQI value observed at this stage serves as prior knowledge to be transmitted to the agent model. Here, a Gaussian process [37] is adopted as the surrogate model, under the assumption that the objective function L(θ) follows a Gaussian process prior:

(17) $$L\left(\theta \right)∼GP\left(\mu \left(\theta \right),k\left(\theta ,\theta^{\prime }\right)\right)$$

where μ(θ) is the mean function, which is set as a constant here, i.e., μ(θ)=c; $k\left(\theta ,\theta^{\prime }\right)$ is the covariance function, and in this paper, we use the Automatic Relevance Determination-Radial Basis Function (ARD-RBF), which has the following expression:

(18) $$k\left(\theta ,\theta^{\prime }\right)=\sigma_{f}^{2}\cdot \exp\left(-\frac{1}{2}{\left(\theta -\theta^{\prime }\right)}^{T}\Lambda^{-1}\left(\theta -\theta^{\prime }\right)\right)$$

where $\sigma_{f}^{2}$ is the signal variance, which controls the output scale of the kernel function; $\Lambda =\mathrm{diag}\left(\ell_{1}^{2},\ell_{2}^{2},\ldots ,\ell_{7}^{2}\right)$ is the ARD length scale.

To ensure appropriate parameter settings for the μ(θ), $\sigma_{f}^{2}$, and Λ, thereby enabling the model to achieve optimal performance, this work adopts the approach of maximizing the marginal log-likelihood (MLL) to train the Gaussian process regression model and determine the optimal hyperparameter configuration. The corresponding objective function is defined as follows:

(19) $$\begin{array}{c} \;\phi^{*}=\underset{\phi }{\arg\max}\log p\left(y|X;\phi \right)\\ \;s.t.\;\log p\left(y|X,\phi \right)=-\frac{1}{2}{\left(y-\mu \right)}^{T}K^{-1}\left(y-\mu \right)-\frac{1}{2}\log\left|K\right|-\frac{n}{2}\log2\pi \\ X={\left[\theta_{1},\theta_{2},\ldots ,\theta_{n}\right]}^{T}\in R^{n\times 7}\\ y={\left[f\left(\theta_{1}\right),f\left(\theta_{2}\right),\ldots ,f\left(\theta_{n}\right)\right]}^{T}\in R^{n\times 1}\\ \mu ={\left[c_{1},c_{2},\ldots ,c_{n}\right]}^{T}\in R^{n\times 1}\\ K_{ij}=k\left(\theta_{i},\theta_{j}\right),\;i,j=1,2,\ldots n \end{array}$$

where ϕ denotes the set of all hyperparameters in the Gaussian process model, and $\phi^{*}$ represents the optimal set that maximizes the marginal log-likelihood (MLL); X is the input sample matrix, in which each row $\theta_{i}$ represents a hyperparameter vector, and *n* denotes the number of observed samples; y is the corresponding output observation vector, μ is the mean vector, and K is the covariance matrix, where $K_{ij}$ denotes the covariance between input samples $\theta_{i}$ and $\theta_{j}$.

After obtaining the Gaussian process surrogate model, the potential of each candidate point is evaluated using an acquisition function based on its predicted mean and uncertainty, and the point with the highest potential value is then selected as the next target for assessment. This work adopts the log expected improvement (LEI) as the acquisition function to evaluate the expected improvement of a candidate point relative to the current optimum on a logarithmic scale, thereby guiding the selection of the next query point. The LEI function can effectively balance exploration in high-uncertainty regions with exploitation of known high-performing regions, and compresses the numerical range of the objective function via logarithmic transformation, thereby enhancing the numerical stability of the optimization process. The analytical expression of the function is provided below:

(20) $$\log\mathrm{EI}\left(\theta \right)=E\left[\max\left(\log\left(f\left(\theta \right)\right)-\log\left(f^{+}\right),0\right)\right]$$

where $f^{+}$ denotes the currently observed optimum of the objective function. After each acquisition and objective function evaluation, the corresponding point is added to the set of observed samples, and the surrogate model is updated accordingly. This iterative process continues to gradually approximate the global optimum until the termination condition is met. Upon completion of the optimization process, the current optimal parameters and their corresponding TMQI values are stored in the task memory of the meta-learning module as historical experience to guide subsequent meta-training. Finally, the optimal parameters are substituted into the method described in Section 4.2 to generate the final tone-mapped image.

## 5. Experimental Results and Discussion

### 5.1. Experimental Details

In the implementation of the Gaussian kernel function, the range of spatial frequencies directly affects the parameter interval of T, which ultimately determines performance. In this work, we take intervals of [−0.5, 0.5] in two directions of spatial frequency, and we divide them equally in different directions based on the resolution of the input image, followed by conversion to polar coordinates and normalization. Based on this, the parameter search space is defined as follows: S∈[0.1,1.0],G∈[0.1,2.5],b∈[0,1],T∈[0.003,0.01],A∈[0.1,10],B∈[0.4,1.0],γ∈[0.1,1.0]. In the meta-learning stage, in order to save memory and computing resources, while ensuring data timeliness and avoiding interference from historical irrelevant information, this paper sets the maximum capacity of the task memory bank to 200. For each meta-training iteration, 50 tasks are sampled and divided into support and query sets in a 1:4 ratio. When the number of tasks in the memory bank exceeds 50, the addition of each new task initiates a clustering operation, maintaining a fixed number of clusters at five. During meta-training, the Adam optimizer is employed, with five iterations in the inner loop, a learning rate of 0.2 for the inner loop, and a learning rate of 0.003 for the outer loop. The mean squared error (MSE) is used as the loss function for both loops. The Bayesian optimization module is implemented using the GPyTorch (1.13) and BoTorch (0.12.0) frameworks. The experimental environment for this paper is shown in Table 1.

### 5.2. Evaluation Indicators

Regarding objective quality evaluation, this study establishes a multidimensional assessment system that comprehensively utilizes TMQI, the Blind Tone Mapping Quality Index (BTMQI) [38], structural fidelity, and naturalness metrics for collaborative analysis. Given the inherent limitations of individual evaluation metrics, reliance on a single standard may introduce assessment bias. Therefore, cross-validation through multiple indicators enables both performance evaluation relative to reference images and independent evaluation of the inherent quality of generated images, ensuring comprehensive and reliable results. Specifically, TMQI measures perceptual consistency through a weighted combination of multi-scale structural fidelity and statistical naturalness. BTMQI, as a reference-free metric, evaluates inherent quality based on the image’s own statistical characteristics. These metrics form a complementary validation mechanism grounded in full-reference and no-reference theoretical foundations, respectively. Structural fidelity assesses the preservation of detailed and edge information, while naturalness (NSS) evaluates whether the image aligns with human visual expectations of natural scenes. Together, they support a comprehensive analysis of algorithmic performance.

1. Structural Fidelity

Structural fidelity quantifies the local structural similarity between a tone-mapped LDR image and its reference HDR image. This metric focuses on local contrast and structural consistency in luminance distribution and is typically implemented using a local similarity formulation similar to SSIM, which computes the mean, variance, and covariance within local neighborhoods to produce a structural similarity index. A global quality score is subsequently obtained for the entire image through spatial weighting or multiscale aggregation. Structural fidelity is a full-reference metric, in which higher scores correspond to a superior preservation of image structures. This study adopts an implementation consistent with prior literature [32], employing an 11 × 11 Gaussian-weighted local window and stabilization constants to maintain numerical stability. Let *x* and *y* denote the local patches of the reference HDR image and the corresponding tone-mapped LDR image, respectively. The local structural fidelity can be defined as follows:

(21) $$S_{\mathrm{local}}\left(x,y\right)=\frac{2{\tilde{\sigma }}_{x}{\tilde{\sigma }}_{y}+C_{1}}{{\tilde{\sigma }}_{x}^{2}+{\tilde{\sigma }}_{y}^{2}+C_{1}}\cdot \frac{\sigma_{xy}+C_{2}}{\sigma_{x}\sigma_{y}+C_{2}}$$

where $\sigma_{x}$ and $\sigma_{y}$ denote the local standard deviations, $\sigma_{xy}$ represents the local cross-correlation, and $\tilde{\sigma }$ denotes the signal strength after nonlinear mapping, used to distinguish between perceptually significant and imperceptible signals; $C_{1}$ and $C_{2}$ are stabilization constants introduced to maintain numerical stability. Subsequently, spatial pooling and scale weighting are performed on local results across multiple scales to derive the global structural fidelity, denoted as *S*. A commonly adopted aggregation formulation is given as follows:

(22) $$S=\sum_{j=1}^{M}w_{j}\cdot \frac{1}{N_{j}}\sum_{i=1}^{N_{j}}S_{\mathrm{local}}^{\left(j\right)}\left(i\right)$$

where *j* denotes the scale index, $N_{j}$ denotes the number of local windows at scale *j*, and $w_{j}$ represents the weight assigned to scale *j*.

2. Natural Scene Statistics (NSS)

Statistical naturalness quantifies the degree to which the luminance statistics of a tone-mapped image conform to the characteristic statistical distributions of natural scenes. Specifically, it analyzes the global or local luminance statistics of an image and estimates their likelihood based on a pre-trained statistical model of natural images to yield a quantitative naturalness score. Statistical naturalness serves as a complementary measure to structural fidelity: even when structural similarity is maintained, an image may still appear perceptually unnatural when its luminance distribution substantially deviates from the typical statistical properties of natural scenes. Higher NSS values generally indicate a closer correspondence to the statistical characteristics of natural scenes. Due to the computational complexity of the process, detailed formulations are omitted here; readers are referred to [32] for a comprehensive description.

3. Tone-Mapped Image Quality Index (TMQI)

TMQI is a comprehensive reference-based metric specifically developed for evaluating tone-mapped image quality. It combines structural fidelity and statistical naturalness to jointly assess both structural preservation and perceptual naturalness. In short, TMQI aggregates these two complementary components, yielding a composite quality score suitable for ranking and comparative evaluation. The computation of TMQI directly depends on these two constituent components. In our implementation, we follow the parameter settings and numerical stabilization strategy recommended in the original paper to ensure consistency and comparability with prior studies. The overall computation is formulated as follows:

(23) $$\mathrm{TMQI}\left(x,y\right)=Q=a\cdot S^{\alpha }+\left(1-a\right)\cdot N^{\beta }$$

where *x* denotes the reference HDR image, and *y* denotes the tone-mapped LDR image under evaluation. *S* denotes structural fidelity, and *N* denotes statistical naturalness. The parameter *a* ∈ [0, 1] serves as a weighting factor, and α, β > 0 control the sensitivity of the two components. To ensure comparability with previous studies, this work follows the fusion strategy and parameter settings recommended in the original publication when computing TMQI.

4. Blind Tone-Mapping Quality Index (BTMQI)

Considering scenarios where the original HDR image is unavailable or cannot be directly utilized as a reference, we additionally incorporate the no-reference perceptual quality prediction metric BTMQI for evaluation purposes. BTMQI operates by extracting multidimensional statistical features—such as luminance distribution, local contrast, and detail texture—from a single LDR image, followed by a prediction of subjective quality scores via a trained regression model, thereby rendering it suitable for blind assessment contexts. In contrast to TMQI, BTMQI does not require a reference HDR image and can furnish an estimation of visual quality under no-reference conditions; consequently, in our result reporting, BTMQI functions as a complementary metric to TMQI, facilitating the evaluation of the algorithm’s potential subjective perceptual performance in engineering deployments. For the specific calculation process, refer to [38].

### 5.3. Comparison of Tone Mapping

To validate the effectiveness and superiority of the proposed algorithm in image tone mapping, this study conducts comparisons with both mainstream and state-of-the-art methods on three publicly available datasets—LVZ-HDR [30], HIG [39], and HSD [40]—comprising a total of 553 HDR images. These datasets encompass a diverse range of complex scenes, including indoor environments, natural landscapes, nighttime scenes, direct sunlight, and water reflections. The compared methods include the following: [30], a full-reference tone-mapping method; [34], an unpaired method; [35,41], reference-free methods; [42], a self-supervised method; and [18,21,23,27,43,44,45,46], traditional methods.

Experimental results are presented in Table 2, Table 3 and Table 4, with the best and second-best scores highlighted in bold and underline, respectively. The proposed method demonstrates substantial overall advantages in performance across all datasets, with highly consistent and complementary results across metrics. Regarding the TMQI metric, the proposed method ranks first on all three datasets, achieving 0.968 on the HIG dataset and surpassing the second-best method (Mujtaba et al. [18] 0.938) by 0.03; on the LVZ-HDR and HSD datasets, it achieved 0.927 and 0.940, representing stable improvements of 0.028 and 0.017 over the next-best results (0.899 and 0.923) and thereby validating the superiority of the algorithm. BTMQI further confirms the advantage from a no-reference perspective: the proposed method achieves the lowest values across all three datasets—2.15 on HIG (a reduction of 0.46 compared to the second-best Mujtaba et al. [18] at 2.61), 2.87 on LVZ-HDR (slightly better than UnCLTMO’s 2.91), and 2.72 on HSD (a reduction of 0.21 compared to the second-best MPS + Yang et al.’s [27] 2.93). The significant reduction with the HIG dataset further supports the conclusions drawn from TMQI.

Among the sub-metrics, structural fidelity reflects the ability to preserve detailed information from the original HDR image. The proposed method ranks first on the HIG dataset with a score of 0.915, achieved the second-best result of 0.901 on the LVZ-HDR dataset (only slightly below UnCLTMO’s 0.918), and scored 0.896 (second-best) on the HSD dataset, demonstrating strong preservation of detail, texture, and edge information. More critically, the proposed method demonstrates marked superiority in the naturalness (NSS) metric: achieving 0.928 on the HIG dataset, far surpassing the second-best Mujtaba’s [18] method 0.749 with an improvement of 0.179, representing a relative gain of approximately 23.9%; on the LVZ-HDR dataset, it achieved 0.703, an improvement of 0.173 over the second-best Mujtaba et al. [18] method (0.530), representing a relative improvement of approximately 32.6%; on the HSD dataset, it reached 0.797, an improvement of 0.150 over the second-best Liang et al. [21] method (0.647), representing a relative improvement of approximately 23.2%. The average absolute improvement across the three datasets was 0.167, with an average relative improvement of approximately 27%. This substantial cross-dataset relative improvement clearly demonstrates that the proposed method does not achieve gains in one dimension at the expense of another. Instead, it comprehensively optimizes perceptual quality, structural preservation, and naturalness. This successfully suppresses artifacts common in existing methods—such as luminance distortion and halos—that degrade natural visual perception. The approach achieves a balanced representation of subjective naturalness, thereby aligning more closely with the holistic perceptual characteristics of the human visual system. This is also a key reason for its superior performance on TMQI and BTMQI. In summary, the proposed method demonstrates distinct advantages over both traditional and deep learning-based approaches.

**Figure 7:**
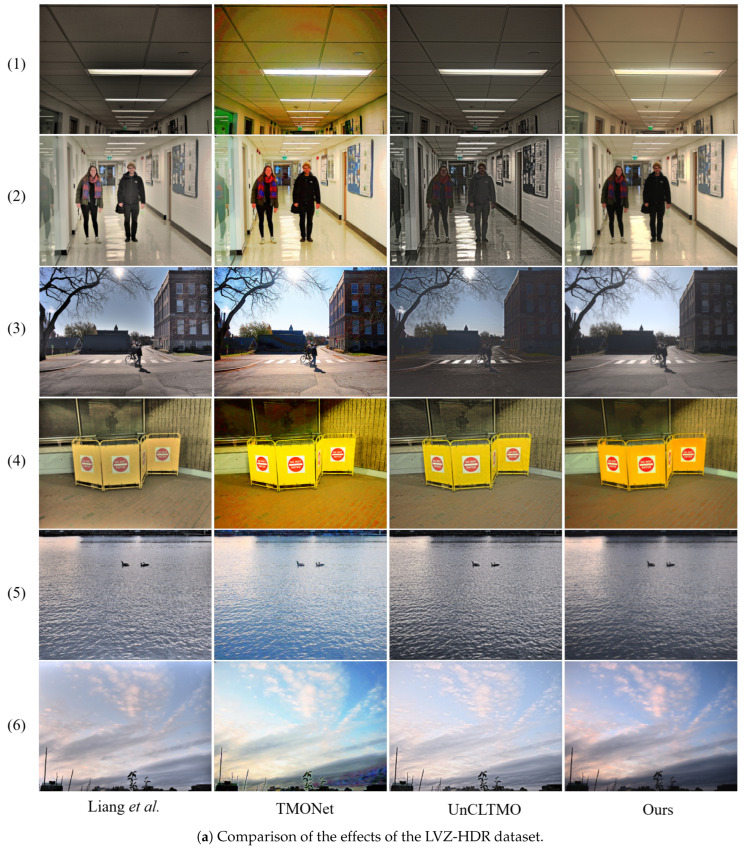

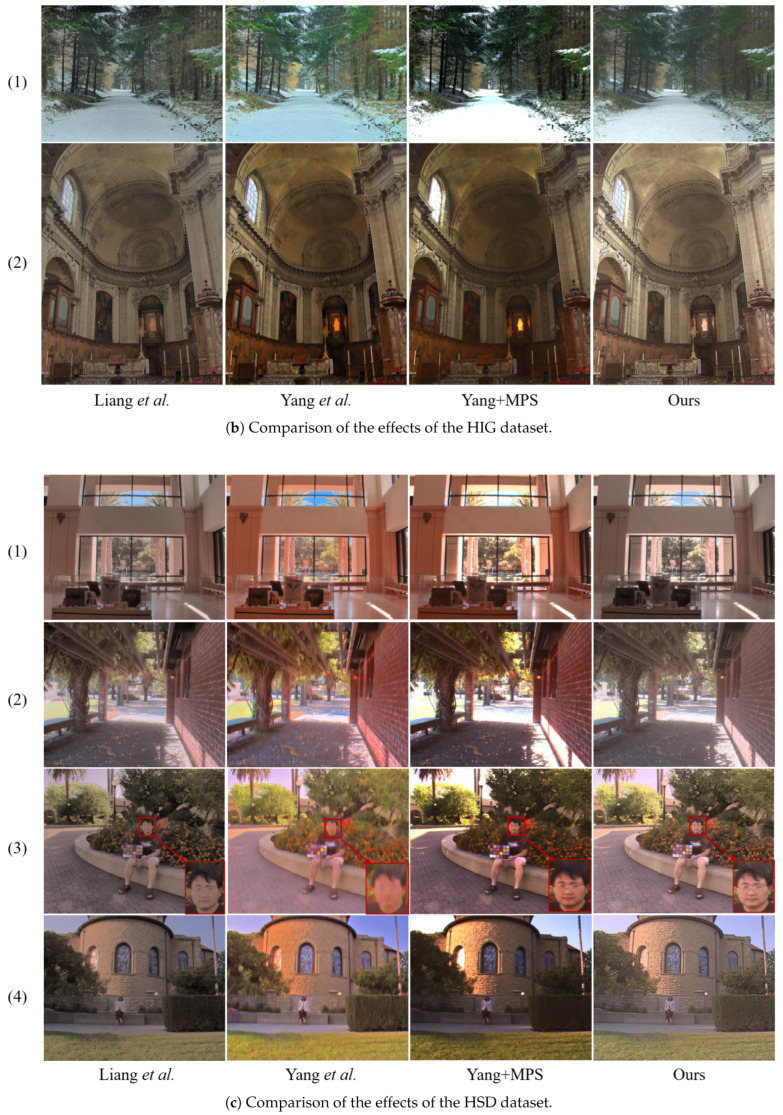
Comparison of visual effects of HDR image tone mapping.

Figure 7 presents a comparison of the results of several mainstream and state-of-the-art tone mapping methods alongside our proposed method across three datasets. Liang et al.’s [21] approach exhibits a significant halo effect in several scenes, evidenced by the formation of large dark areas at the bright-dark junctions. This is primarily because the method fails to adequately handle edge gradients during dynamic range compression, thereby forming dark contours due to unnatural suppression of pixel values at the edges of bright regions. Although TMONet effectively avoids the halo effect, it introduces pronounced color banding artifacts in the grayscale transition regions (Figure 7a, first row of images) while also producing images with high saturation and false colors. Noticeable overexposure is also observed in scenes with intense sunlight (Figure 7a, third row). UnCLTMO performs better in managing halo effects, artifacts, and saturation; however, localized issues of overexposure and underexposure remain in certain backlit and high-contrast scenes, indicating that the dynamic range compression is still insufficient. In addition, the method exhibits noticeable oversharpening, introducing increased noise and resulting in significant texture distortion (Figure 7a, second and third rows). The method of Yang et al. [45] demonstrates strong dynamic range compression for HDR images, but the overall saturation of the output image is high, the tone mapping appears unnatural, and over-enhancement in some scenes leads to contrast distortion (Figure 7b, first row). Moreover, the method also results in a loss of image details, with high-frequency features poorly preserved (e.g., the face area in Figure 7c, third row). Qiu et al. [27] improved upon Yang’s [45] method by employing multi-peak curves and accordingly proposed the Yang + MPS method. While this approach mitigates contrast distortion and detail loss, it introduces the issue of overexposure in localized highlight regions. In addition, by carefully examining the objective evaluation data alongside the tone-mapped output images, one can readily observe that visual distortions—such as anomalous brightness in the sky, irregular luminance in shadowed areas, excessive saturation, or overall color shifts—are directly tied to the lack of naturalness. Most existing algorithms, however, pursue higher structural fidelity to enhance TMQI, primarily because NSS is non-differentiable and lacks a fully corresponding surrogate loss. This often results in over-enhanced local contrast, producing artifacts in highlights, unnaturally harsh brightening of dark regions that flattens depth, and jarring transitions between adjacent areas. In contrast, the method proposed in this paper employs Bayesian optimization to compensate for the absence of the NSS term in gradient-based approaches. By balancing fidelity and NSS, it achieves smoother, more natural grayscale transitions, preserves bright-region details with clarity, effectively enhances shadowed areas, and faithfully reproduces image textures while maintaining appropriate contrast. Overall, the LDR images generated via our method exhibit superior visual quality and better align with human visual perception.

### 5.4. Ablation Experiments

Ablation experiments are conducted to analyze the contribution of different modules to tone mapping performance and to assess the effectiveness of each component of the proposed algorithm. Specifically, the experiments include the following components: (i) evaluating the performance of the phase modulation module by using only four parameters—*S*, *G*, *b*, and *T*; (ii) evaluating the performance of the Stevens power law and saturation adjustment module by using only three parameters—*A*, *B*, and γ; and (iii) comparing meta-learning-guided Bayesian optimization with randomly initialized classical Bayesian optimization under different iteration settings, and analyzing its impact on the convergence speed.

Table 5 presents the results of the first two ablation experiments, indicating that neither module achieves optimal performance in isolation. This is because the phase modulation in virtual diffraction, although capable of achieving dynamic range compression to a certain extent, has a limited ability to adjust the global luminance distribution. Its primary advantage lies in the precise enhancement of image details and contrast, which effectively mitigates contrast distortion in the output image. In comparison, the model based on Stevens’s power law can significantly adjust the image brightness distribution through nonlinear transformation but exhibits notable limitations in local detail restoration and contrast preservation. Therefore, combining both modules allows them to leverage their complementary strengths, thereby maximizing the preservation of image details while achieving effective dynamic range compression.

Moreover, the superiority of meta-learning can only be demonstrated when the image data reaches a certain scale. However, the dataset size of HIG is relatively small and does not independently meet the conditions required for the final ablation experiment. Therefore, this paper combines the HIG and HSD datasets for experimentation. The experimental results are presented in Table 6, where ✓ denotes the application of meta-learning and ✗ indicates its absence.

The experimental results demonstrate that meta-learning is particularly effective in guiding the initial stages of the optimization process. For instance, on the LVZ-HDR dataset, a TMQI score of 0.874 can be achieved with only a single evaluation when meta-learning is employed, whereas the non-meta-learning approach requires approximately five iterations to reach a comparable performance. Similarly, on the combined HIG + HSD dataset, a TMQI score of 0.901 is attained in the first evaluation with meta-learning, representing an almost 5× speedup compared to the non-meta-learning approach. This observation suggests that meta-learning can rapidly capture the macro-structure of the objective function with less data, providing a high-quality initialization for subsequent Bayesian optimization, which is significantly better than random or uniform initialization strategies and enables up to five-fold acceleration in convergence. During the iterations from the 5th to the 10th, the acceleration performance remains within the range of 2× to 5×, although it slightly declines. Specifically, on the LVZ-HDR dataset, the meta-learning approach achieves a speedup of approximately 2× at the 5th iteration and 3× at the 10th; similarly, on the HIG + HSD dataset, it achieves a speedup of over 5× at the 5th iteration and 3× at the 10th. This indicates that meta-learning exerts a sustained positive effect on sample utilization efficiency in the early stages, significantly shortening the optimization path and enabling TMQI to reach moderate-to-high levels rapidly. When the number of iterations is further increased to 30 and above, the TMQI scores of both strategies converge; the acceleration effect diminishes, and the optimization process tends toward convergence. These findings confirm that initializing the surrogate model with historical knowledge distilled through meta-learning substantially improves the efficiency of initial sample usage, effectively alleviates the cold-start bottleneck of traditional Bayesian optimization—often sensitive to initial samples and prone to inefficient exploration in early stages—and enhances the overall convergence performance.

**Figure 8:**
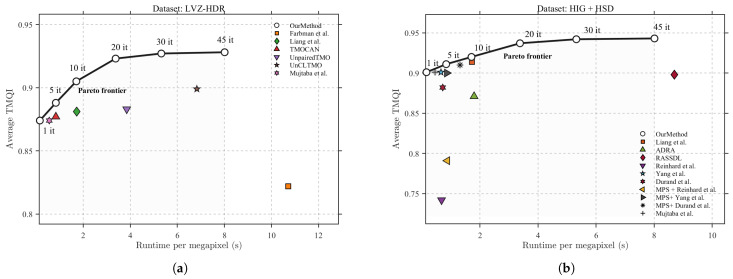
The Pareto curve of time and quality. (**a**) Pareto curve of the LVZ-HDR dataset. (**b**) Pareto curve of the LVZ-HDR dataset.

In practical tone-mapping applications, perceptual quality and computational cost represent inherently conflicting objectives. To quantify this trade-off, Figure 8 illustrates a Pareto-front analysis in which the average TMQI is plotted against the runtime per megapixel. Each data point represents a specific method evaluated under the experimental conditions detailed in Table 1, whereas a series of white circular markers corresponds to our framework under varying optimization budgets, thereby forming an empirical Pareto frontier.

Across all three datasets, the proposed framework distinctly delineates the Pareto-optimal boundary, positioned along the upper-left envelope of all evaluated methods. Within the low-runtime regime, our method achieves TMQI values exceeding 0.90 and surpasses the state-of-the-art traditional and deep learning-based baseline methods within 10 iterations, including Liang et al. [21], TMOCAN [41], and ADRA [43]. As the optimization budget increases, the curve exhibits a monotonic upward trend with diminishing marginal gains, ultimately converging smoothly to a TMQI of less than 0.95 at 5.2 s per megapixel. This saturation behavior indicates an efficient convergence toward a near-optimal perceptual–efficiency equilibrium, where additional computation yields only marginal quality enhancement.

In contrast, competing operators occupy dominated regions of the Pareto plane—either requiring substantially longer runtimes for similar quality (e.g., UnCLTMO [35]) or yielding lower TMQI despite faster inference (e.g., Yang et al. [45], Durand et al. [46], and Mujtaba et al. [18]). The consistent Pareto dominance across both benchmark groups confirms that our meta-guided Bayesian optimization effectively balances quality maximization and computational efficiency within a unified framework. Furthermore, the smooth progression of the iteration-wise results indicates that the proposed method offers controllable quality–efficiency scaling, allowing it to adapt its performance according to the available computational budget in HDR imaging applications.

Furthermore, with the increasing adoption of Bayesian optimization in machine learning and the ongoing refinement of GPyTorch and BoTorch, their runtime performance on GPUs is anticipated to improve further. Therefore, this method provides a robust technical foundation for achieving real-time processing in future applications.

### 5.5. Robustness Analysis

Noise interference is a pervasive and inevitable challenge in HDR image acquisition, processing, and display. For instance, additive Gaussian noise arising from sensor thermal fluctuations or impulse noise introduced via transmission errors can perturb the image’s spectral distribution, compromising the accuracy of virtual diffraction phase modulation and psychophysical brightness perception models, and ultimately degrading the overall tone-mapping quality. To evaluate the robustness and practical applicability of the proposed framework under noisy conditions, this section conducts a quantitative analysis by introducing minor noise perturbations into a benchmark dataset.

Specifically, a relatively conservative evaluation strategy is adopted, in which 50 representative HDR images encompassing a diverse range of scenes are randomly selected from the dataset. Prior to noise injection, the images are normalized, such that pixel values fall within the range [0, 1]. Owing to the extremely wide dynamic range of HDR images, the vast majority of pixels after normalization assume very low values, while only a small proportion of bright-region pixels approach unity. This leads to a markedly non-uniform distribution of pixel intensities. To closely emulate the subtle interference of real-world noise while preventing it from overwhelming the original image content, Gaussian noise with a standard deviation (σ) ranging from 0.001 to 0.01 and impulse noise with a density between 0.1% and 1% were introduced. The optimal parameters were determined through meta-guided Bayesian optimization conducted on the clean HDR images. These parameters were subsequently applied to the noisy counterparts of the same images, and the relative degradation of the tone-mapped outputs was quantified based on the TMQI metric. This approach isolates and evaluates the intrinsic robustness of the core tone-mapping operations—including virtual diffraction phase modulation and Stevens’s power-law adjustment—against noise, without necessitating adaptive modifications during the optimization procedure. The corresponding results are presented in Table 7.

Experimental results demonstrate that the proposed method exhibits remarkable robustness against both Gaussian and impulse noise. As noise intensity increases, the TMQI score shows only a gradual and controlled decline. Specifically, when Gaussian noise with a standard deviation below 0.004 is introduced, the average TMQI decreases slightly from 0.935 to 0.919—representing a relative reduction of only 1.65%. Even at σ = 0.006, the TMQI remains above 0.908. A noticeable degradation is observed only when σ increases to 0.01, where the noise becomes perceptually evident. A similar trend is found for impulse noise: at a noise density of 0.8%, the TMQI decreases by less than 3%, and even at 1% the decrease remains within 6.4%, confirming the algorithm’s strong resilience to noise interference.

This robustness arises primarily from three intrinsic aspects of the algorithmic design. First, through low-pass spectral phase modulation, the framework selectively processes low-frequency components, while typical image noise—predominantly concentrated in the high-frequency range—remains largely unaffected by phase perturbations. This frequency-domain design naturally introduces a smoothing effect in the spatial domain, forming an inherent noise suppression mechanism. Second, a two-stage noise control strategy is embedded within the tone-mapping framework. The bias term *b* enhances numerical stability and suppresses small-scale noise perturbations, while the nonlinear transformation of the arctangent function can compress the signal dynamic range and suppresses spurious fluctuations. Finally, the framework’s reliance on phase information—rather than intensity—provides an additional layer of robustness, as phase representations are intrinsically less sensitive to additive noise and preserve fine structural details even under degraded conditions.

In summary, the synergistic integration of low-frequency phase modulation, dual-stage spatial-domain suppression, and phase-based representation establishes a solid foundation for the method’s stability in noisy and complex real-world environments. The experimental data fully validate both the theoretical soundness and practical effectiveness of this design. This result demonstrates the considerable application potential of the proposed method across various domains, including film post-production, astrophotography, satellite remote sensing, high-end commercial photography, premium advertising, and digital signage.

## 6. Conclusions

In this work, we present a novel image tone-mapping framework that integrates virtual light-field diffraction, a psychophysical luminance model, Bayesian optimization, and task-distribution-oriented meta-learning. By abstracting virtual diffraction as a phase modulation operator in the frequency domain and coupling it with Stevens’s power law to simulate the nonlinear luminance perception of the human visual system, our method enables precise control over both local details and global brightness distribution. The Bayesian optimization module, guided by meta-learned initialization, facilitates efficient optimization of the multi-parameter tone mapping model and effectively overcomes the limitations of random initialization. Because Bayesian optimization does not rely on gradients, it can directly optimize TMQI, thereby compensating for the deficiencies in NSS arising from gradient-based methods, achieving a balance between fidelity and naturalness, and substantially enhancing visual perception. Experiments demonstrate that the proposed framework outperforms state-of-the-art tone mapping methods in overall performance. It consistently achieves the highest TMQI, BTMQI, and NSS scores while balancing dynamic range compression, detail preservation, and natural appearance, with an average improvement of 27% in NSS. Simultaneously, it attains the highest or second-highest fidelity scores and generates visually pleasing LDR outputs. Ablation studies further validate the complementary roles of the phase modulation and Stevens’s power law modules and highlight the accelerated convergence attained through meta-learning-guided Bayesian optimization, which is two- to five-fold faster. This method consistently lies on the Pareto frontier, demonstrating an optimal balance between performance and computational time.

## Figures and Tables

**Table 1:** Experimental environment configuration.

Configuration Item	Value
CPU model	Intel(R) Core(TM) i7-12700F @ 2.10 GHz
Memory	64 G
GPU model	NVIDIA RTX 3060
Graphics memory	12 G

**Table 2:** Comparison results on the LVZ-HDR Dataset. (The upward arrow in the table denotes that higher values indicate better performance, whereas the downward arrow signifies that lower values are preferable. This convention is consistently applied to all arrows in the tables below.)

Method	Dataset: LVZ-HDR			
	BTMQI ↓	TMQI ↑	Fidelity ↑	NSS ↑
Farbman et al. [23]	5.15	0.822	0.674	0.451
Liang et al. [21]	3.45	0.881	0.855	0.506
TMONet [30]	3.44	0.873	0.831	0.475
TMOCAN [41]	3.32	0.877	-	-
UnpairedTMO [34]	3.03	0.883	0.898	0.474
UnCLTMO [35]	2.91	0.899	**0.918**	0.503
Mujtaba et al. [18]	3.04	0.874	0.858	0.530
**Ours**	**2.87**	**0.927**	0.901	**0.703**

**Table 3:** Comparison results on the HIG Datasets.

Method	Dataset: HIG			
	BTMQI ↓	TMQI ↑	Fidelity ↑	NSS ↑
Liang et al. [21]	2.91	0.903	0.904	0.557
ADRA [43]	4.12	0.852	0.866	0.351
RASSDL [42]	-	0.912	0.877	0.646
Reinhard et al. [44]	6.99	0.623	0.504	0.004
Yang et al. [45]	3.91	0.867	0.854	0.442
Durand et al. [46]	4.39	0.840	0.865	0.283
MPS + Reinhard et al. [27]	6.87	0.671	0.546	0.120
MPS + Yang et al. [27]	3.23	0.904	0.851	0.632
MPS + Durand et al. [27]	2.98	0.904	0.912	0.542
Mujtaba et al. [18]	2.61	0.938	0.906	0.749
**Ours**	**2.15**	**0.968**	**0.915**	**0.928**

**Table 4:** Comparison results on the HSD datasets.

Method	Dataset: HSD			
	BTMQI ↓	TMQI ↑	Fidelity ↑	NSS ↑
Liang et al. [21]	3.01	0.916	0.889	0.647
ADRA [43]	4.43	0.874	0.831	0.512
RASSDL [42]	-	0.897	0.863	0.583
Reinhard et al. [44]	5.35	0.756	0.819	0.004
Yang et al. [45]	3.47	0.894	0.860	0.572
Durand et al. [46]	3.75	0.887	0.893	0.494
MPS + Reinhard et al. [27]	4.94	0.805	0.803	0.252
MPS + Yang et al. [27]	2.93	0.908	0.869	0.640
MPS + Durand et al. [27]	2.97	0.923	**0.912**	0.638
Mujtaba et al. [18]	3.03	0.902	0.842	0.631
**Ours**	**2.72**	**0.940**	0.896	**0.797**

**Table 5:** Comparative results of ablation experiments (i), (ii).

Method	TMQI ↑		
	LVZ-HDR	HIG	HSD
(i)	0.888	0.824	0.883
(ii)	0.898	0.874	0.918
**Combined**	**0.927**	**0.968**	**0.940**

**Table 6:** Comparative results of the ablation experiment (iii).

Iterations	TMQI ↑				
	LVZ-HDR			HIG + HSD	
	✓	✗		✓	✗
1	0.874	0.862		0.901	0.896
5	0.888	0.873		0.911	0.902
10	0.905	0.898		0.920	0.908
30	0.927	0.914		0.942	0.930

**Table 7:** Quantitative results of the noise robustness evaluation.

Noise Type	Level (Std. (σ)/Density)	TMQI ↑	Degradation (%)
No noise	0	0.935	0
	0.002	0.926	1.01
	0.004	0.919	1.65
Gaussian noise	0.006	0.908	2.86
	0.008	0.889	4.91
	0.010	0.867	7.24
	0.20%	0.930	0.56
	0.40%	0.921	1.47
Impulse noise	0.60%	0.915	2.13
	0.80%	0.907	2.96
	1.00%	0.875	6.38

## Data Availability

All the data used in this study are based on publicly available data sets and can be found in Refs. [30,39,40]. Requests for further information and resources should be directed to and will be fulfilled by the lead contact, Xinyue Mao (maoxy@ccxida.com).
